# *X1AKAP1* Deficiency Attenuates Diet‐Induced Obesity and Insulin Resistance by Promoting Fatty Acid Oxidation and Thermogenesis in Brown Adipocytes

**DOI:** 10.1002/advs.202204669

**Published:** 2022-10-05

**Authors:** Lele Ji, Ya Zhao, Linjie He, Jing Zhao, Tian Gao, Fengzhou Liu, Bingchao Qi, Fei Kang, Gang Wang, Yilin Zhao, Haitao Guo, Yuanfang He, Fei Li, Qichao Huang, Jinliang Xing

*Adv. Sci*. **2021**, *8*, 2002794

DOI: 10.1002/advs.202002794

In the original published article, the previous forward and reverse primers of *Cidea* in Table S1 (Supporting Information) were reversed. Now these two primers of *Cidea* have been exchanged. Please find the correct Table S1 below.

**Table S1:** The sequence of Primers

Gene	Forward (5′‐3′)	Reverse (5′‐3′)
*AKAP1* (Mouse)	GTTGCCCGGAATGCTGG	TGGGGAGGTTGCCTGAGGA
*36b4* (Mouse)	GCAGACAACGTGGGCTCCAAGCAGAT	GGTCCTCCTTGGTGAACACGAAGCCC
*UCP1* (Mouse)	GGCATTCAGAGGCAAATCAGCT	CAATGAACACTGCCACACCTC
*Cidea* (Mouse)	GCCGTGTTAAGGAATCTGCTG	TGCTCTTCTGTATCGCCCAGT
*Pgc1a* (Mouse)	AATACCGCAAAGAGCACGAG	ACCAACGTAAATCACACGGC
*Ap2* (Mouse)	ACACCGAGATTTCCTTCAAACTG	CCATCTAGGGTTATGATGCTCTTCA
*Adiponectin* (Mouse)	GCACTGGCAAGTTCTACTGCAA	GTAGGTGAAGAGAACGGCCTTGT
*Dio2* (Mouse)	CAGTGTGGTGCACGTCTCCAATC	TGAACCAAAGTTGACCACCAG
*Prdm16* (Mouse)	CAGCACGGTGAAGCCATTC	GCGTGCATCCGCTTGTG
*PPARγ* (Mouse)	TGTCGGTTTCAGAAGTGCCTTG	TTCAGCTGGTCGATATCACTGGAG
*β‐actin* (Mouse)	AGCCATGTACGTAGCCATCCA	TCTCCGGAGTCCATCACAATG
*AKAP1* (Human)	AGCCGAGGAGCTGGTGTAAT	GGGAGAGGTTCCTTGATGGC
*36b4* (Human)	TCGTGGAAGTGACATCGTCTTT	CTGTCTTCCCTGGGCATCA

The authors apologize for any inconvenience caused.

